# How are countries planning for costs of nutrition data and information systems?

**DOI:** 10.12688/gatesopenres.13145.1

**Published:** 2020-06-16

**Authors:** Renee Manorat, Yashodhara Rana, Kyle Borces, Laura Becker, Augustin Flory

**Affiliations:** 1 Independent Researcher, Washington, District of Columbia, USA; 2 Results for Development, Washington, District of Columbia, 20036, USA; 3 Independent Researcher, Seltjarnarnes, Iceland

**Keywords:** Nutrition information system, Nutrition data and information system

## Abstract

**Background:** The first Global Nutrition Report in 2014 called for a “data revolution” in nutrition, so that countries have the latest data to set priorities and monitor progress. Integral to this revolution is understanding how countries are investing in the data, systems and capacity required to support decision-making around nutrition, i.e. their nutrition data and information system (NDIS).

**Methods:** For this reason, our team conducted a desk review of national nutrition plans for 58 Scaling Up Nutrition (SUN) countries to better understand how countries are planning for and estimating the costs of their NDIS.

**Results:** We found that of the SUN national nutrition plans that are publicly accessible, not all are costed and less than half of these have explicit data and monitoring and evaluation (M&E) sections. Of the 19 national plans that had costed data and M&E sections, our initial estimates show costs for data systems ranged from 0.1%-12.8% of total plan costs with limited information on data system components.

**Conclusions:** There is an imminent need for more comprehensive and strategic approaches – including the planning for and financing of – NDIS in countries.

## Introduction

**Figure 1.**
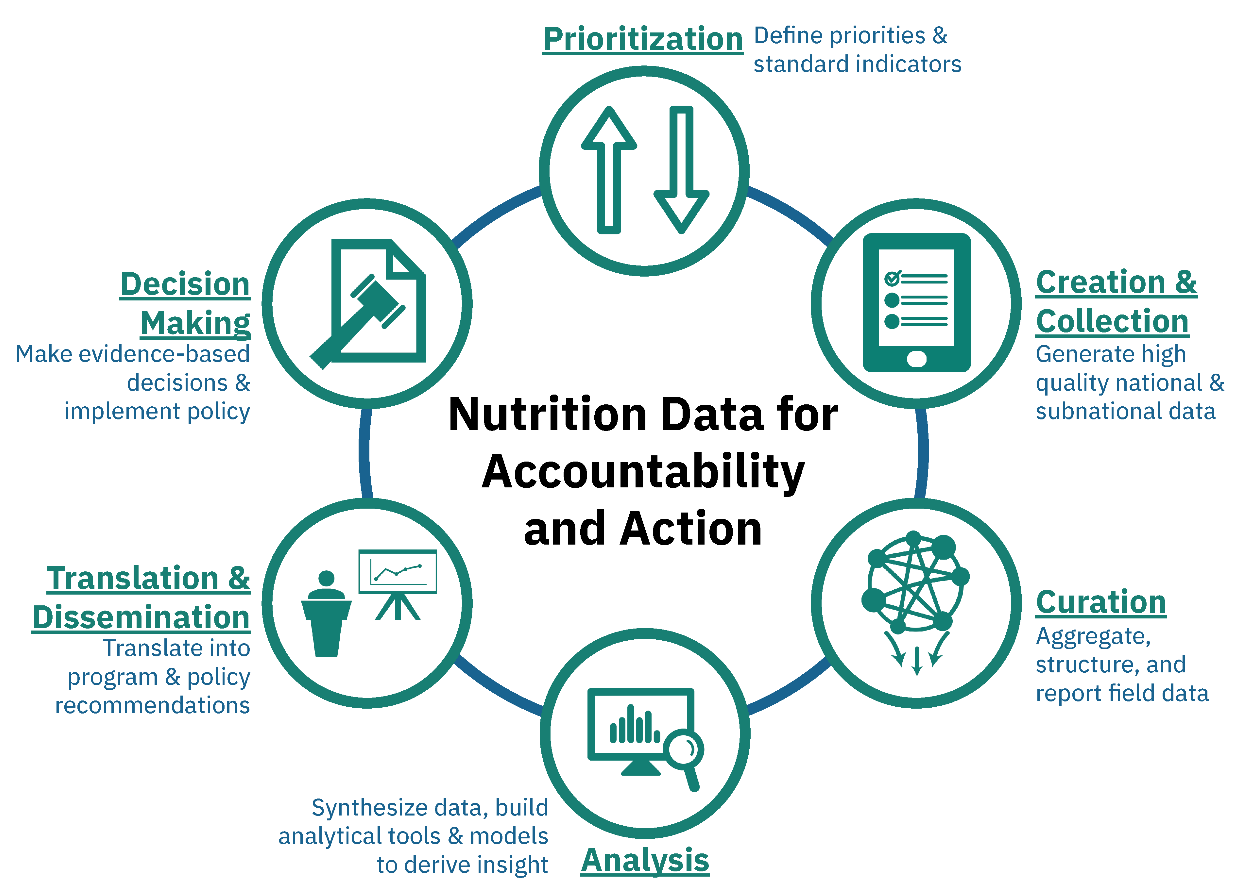
Nutrition Data Value Chain. Adapted from Piwoz *et al*., 2019.

With preparations underway for the Nutrition for Growth (N4G) Summit, the global nutrition community is examining its prior commitments, progress made to date, and outlining the path forward for achieving the World Health Assembly (WHA) global nutrition targets by 2025. There is increasing recognition that the way forward will require higher quality and timely data and investments in all elements of the nutrition data value chain ( Figure 1). Not surprisingly, the 2018 Global Nutrition Report called for increased prioritization and investment in nutrition data recognizing that progress is not possible if we cannot identify where action is most needed ( Development Initiatives, 2018).

Despite this recognition, there is limited guidance for countries regarding how to invest in their NDIS. SUN’s checklist for the development of national nutrition plans includes a monitoring and evaluation framework. More specifically, the framework recommends a multisectoral nutrition information platform to support analysis, knowledge management, learning and communication. This checklist, however, does not provide further guidance regarding how countries can begin to plan, cost and implement a NDIS ( Scaling Up Nutrition, 2016). UNICEF with support from the WHO-UNICEF Technical Expert Advisory group on nutrition Monitoring (TEAM) is currently developing a report to meet country demand for more support in planning and implementation of nutrition information systems ( WHO, 2015).

As country-level NDIS is critical for countries to assess progress towards achieving targets, it is important to understand how countries are planning for and costing NDIS. An improved understanding of countries’ approach can help in the identification of best practices and key gaps that may complement the work being supported by WHO-UNICEF TEAM. Towards this end, our team conducted a review of national nutrition plans for 58 SUN countries to better understand how countries are planning for and estimating the costs of their NDIS ( Manorat *et al*., 2019).

## Methods

**Figure 2.**
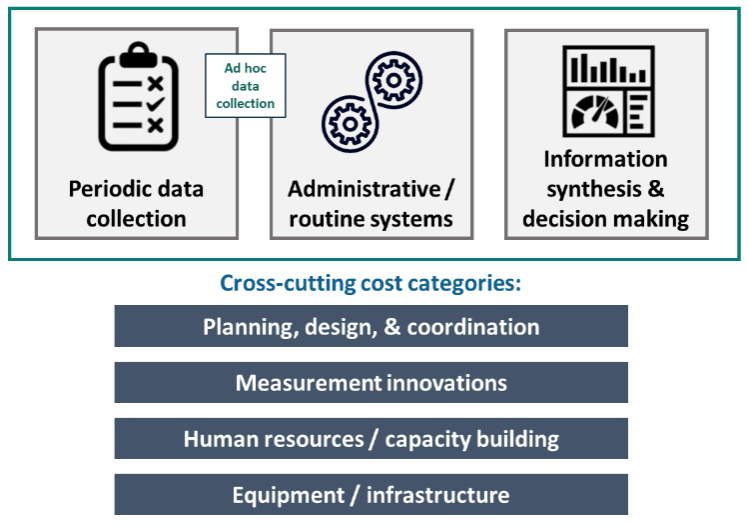
DataDENT framework on the major costs behind nutrition data systems.

For this study, the team accessed the most current national nutrition plans that were publicly available or made available to the team by the SUN Secretariat. The plans that were consulted for this analysis are noted in *Extended data*, Appendix 3 ( Manorat *et al*., 2020). To ensure our team used a consistent approach to review the national nutrition plans, obtained either online or through the SUN Secretariat, we used a framework developed by DataDENT ( Figure 2) that outlines the major cost components needed for establishing and maintaining NDIS. The framework was refined with select country participants from the National Information Platforms for Nutrition (NIPN) Global Gathering in May 2019 and in consultation with stakeholders from Nutrition International (NI), Maximising the Quality of Scaling Up Nutrition Plus (MQSUN+), and the Bill & Melinda Gates Foundation. Please refer to *Extended data*, Appendix 1 ( Manorat *et al*., 2020) for the full list of stakeholders consulted.

Briefly, the framework consists of three main components followed by cross-cutting components — (i) *Periodic data collection* includes data collection activities conducted on a periodic basis, primarily large-scale household surveys conducted annually or every 3–5 years; (ii) *Administrative/routine systems* involves the development, implementation, and data quality assurance of routine management information systems (MIS); (iii) *Information synthesis and decision-making* pertains to the collation, analysis, visualization, and dissemination of nutrition data. In addition to these three main components, we also recognize that there can be a fourth “ad hoc” category that straddles between periodic and routine data collection. This category can include nutrition assessments during emergencies, for instance.

The cross-cutting categories include *planning, design, and coordination* which involves the development of overarching plans for NDIS or monitoring frameworks. *Human resources and capacity building* category includes costs associated with the salaries, training and/or sensitizing people who maintain data systems, analyze data or use the information. *Equipment and infrastructure* describes the infrastructure, supply, and transportation costs for maintaining the information system. Finally, the *measurement innovations* category involves any new tools or processes for collecting, monitoring, and evaluating nutrition data. In *Extended data*, Appendix 2 ( Manorat *et al*., 2020), we have elaborated on these categories and sub-categories along with providing examples drawn from our review.

### Examination and analysis of national nutrition plans

**Figure 3.**
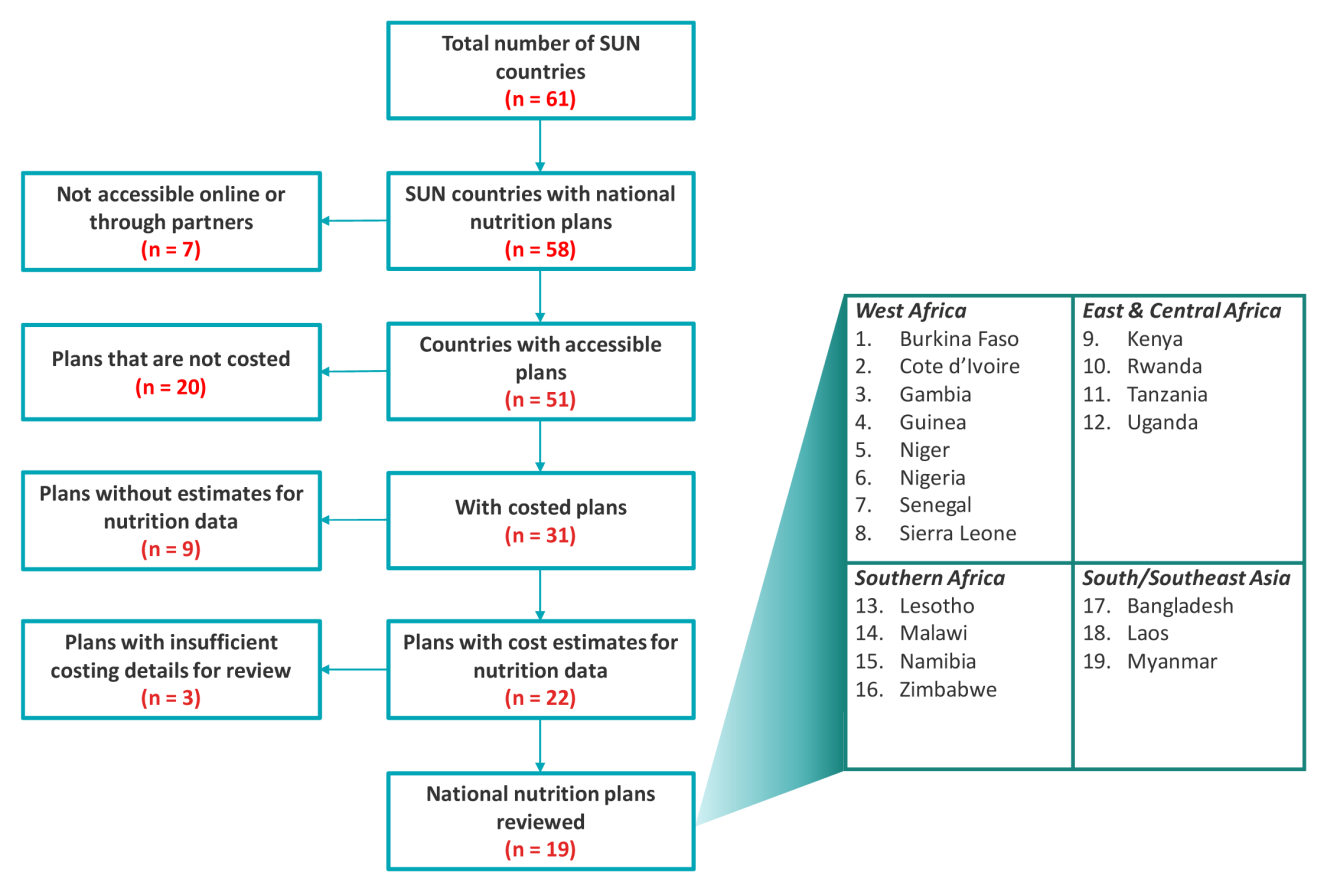
Breakdown of the review process.

Using this framework, a team of two reviewers examined national nutrition plans for 58 SUN countries to understand how they are currently costing for NDIS activities. A total of 31 countries had costed plans that were accessible for our review. Of these costed plans, we found 22 plans that costed for NDIS activities, but two of these plans had insufficient costing details i.e. monitoring and evaluation costs were bucketed with other objectives and so disaggregation was not possible and one plan had outlier cost estimates. In Figure 3, we briefly summarize the process followed for the review.

We conducted an in-depth analysis of 19 national nutrition plans where we extracted the relevant line items that pertained to monitoring, evaluation and data, categorized them according to the framework and then summed up the costs to create an overall cost estimate for NDIS activities. Please refer to the *Underlying data* ( Manorat *et al*., 2020), which provides these details. Given that the desk review is subjective by nature, we took measures to improve confidence in our findings. We developed a codebook prior to conducting the analysis and had two reviewers check each other’s coding. A group of three experts, each with more than 10 years of experience on nutrition and nutrition data-related issues, weighed in cases where it was unclear how best to code cost buckets against the NDIS framework. The team also validated the approach through consultations with SUN stakeholders in Uganda and Vietnam and technical assistance providers from Kenya.

Two key limitations are important to note. First, this research does not intend to provide a comprehensive reflection of a country’s investments in NDIS, but rather focuses on how countries are costing for NDIS specifically in their national nutrition plans. Second, our analysis is limited to the level of reporting available in each plan. For instance, if a country costed for implementing national nutrition surveys or conducting data quality audits of its routine health information system, but then reported them in the plan under one line item for “developing the nutrition information system” then we would not be able to disaggregate this data.

## Results

### The status quo: Inclusion of NDIS costs in national nutrition plans

**Figure 4.**
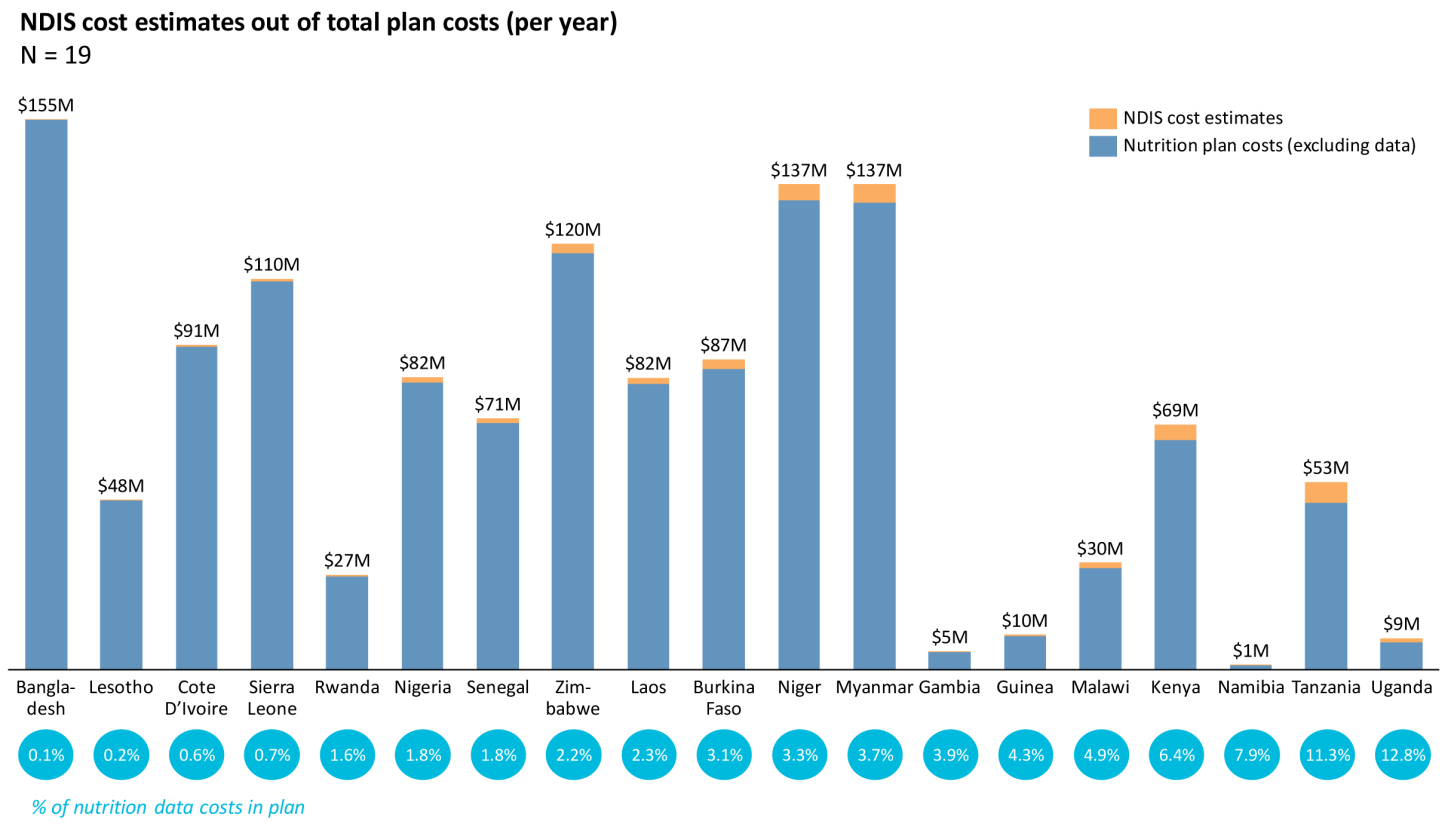
NDIS cost estimates out of total plan costs.

We found that approximately one-third of SUN countries have costed plans with data and M&E sections. Of these plans, we found that data systems costs ranged from 0.1% to 12.8% of total budget costs ( Figure 4). Whereas some countries integrated data-related activities across objectives (e.g., Cote d’Ivoire), others had specific line items for nutrition M&E activities (e.g., Uganda).

**Figure 5.**
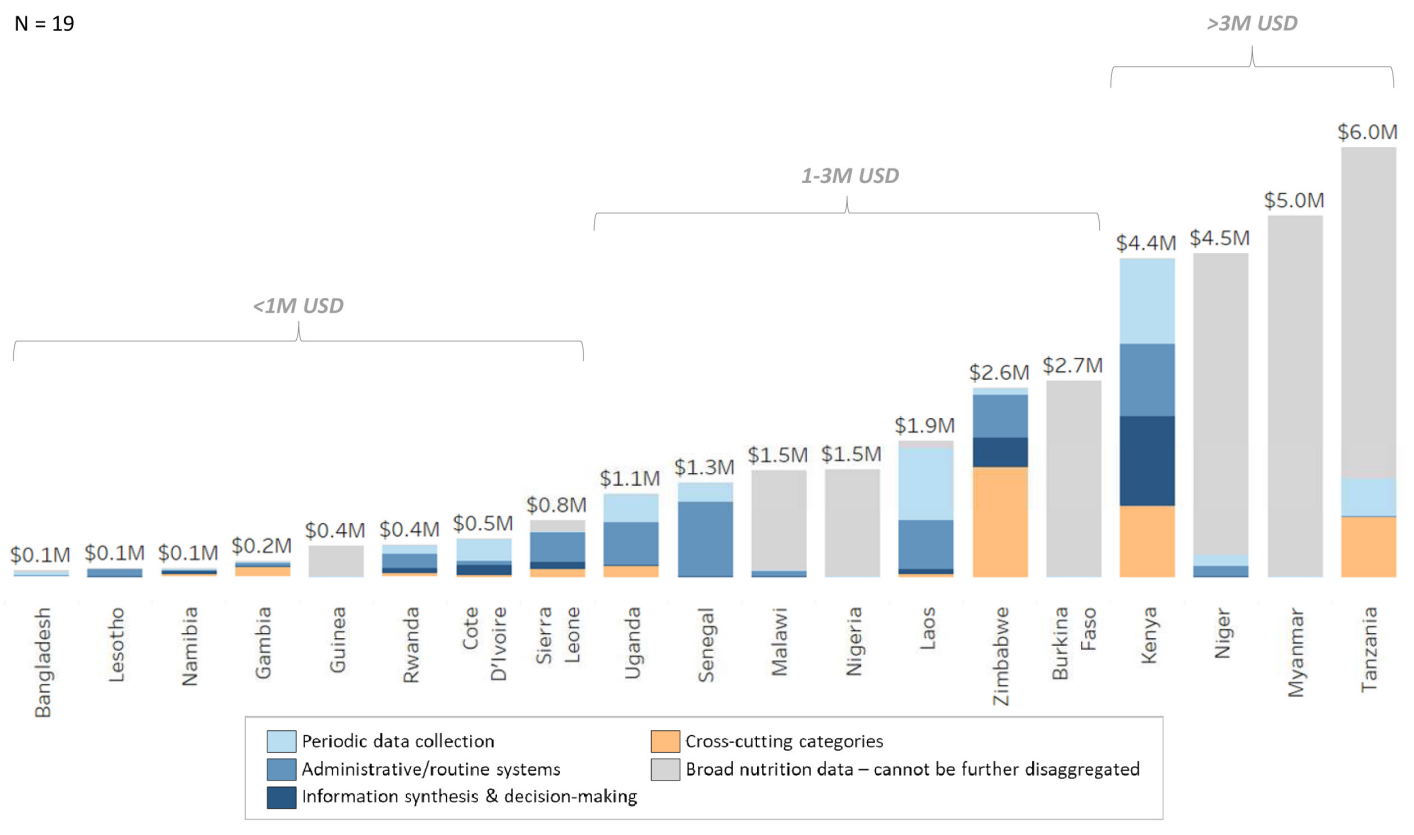
Annual NDIS cost estimates by framework components.

In general, we found that costed plans included limited information on costs of nutrition data and M&E systems beyond a single or limited number of high-level item lines. As can be seen in Figure 5, we have included the *broad nutrition data* category in several countries since we could not further disaggregate the budget lines. As an example, Burkina Faso’s national nutrition plan included a budget line that notes “the nutrition monitoring and evaluation system is improved”. Additionally, Myanmar’s national plan has one costed line item with a description for “monitoring and evaluation”. In both cases, these are very broad activities that could relate to several components of the NDIS framework.

Among the plans we reviewed, we found that the most commonly costed components were periodic data collection, administrative/routine systems and capacity building. On the other hand, there was limited mention of other components critical to the development and maintenance of data systems. Only a few countries included costs for information synthesis and decision-making such as the costs for annual review meetings in Zimbabwe or developing new scorecards and dashboards to visualize nutrition data in Kenya. Countries such as Senegal elaborated more on periodic or administrative/routine data collection activities and had limited mention of line items on the latter half of the nutrition value chain (see Figure 1) that are as critical to ensure the effective use of data to support decision-making. It may be the case that costs associated with the synthesis and analysis of critical nutrition data such as from the Demographic and Health Surveys (DHS) is often paid for by donors and hence not budgeted by countries or that the lack of specificity about assumptions for costing may mean that information analysis and syntheses costs could have been included within the periodic and administrative costs buckets.

Likewise, cross-cutting categories such as planning, design and coordination, measurement innovations and equipment/infrastructure had the least amount of costing information available. Only one plan included measurement innovations, and a few plans budgeted for equipment and supplies. In addition, only four countries had budgeted for resource tracking which is a critical activity that countries need to assess and secure funding towards the implementation of their nutrition national plans.

### Positive outlier: Kenya’s National Nutrition Plan

While our review revealed several gaps, we also found a few countries with more detailed cost estimates for NDIS in their national nutrition plans. Of the costed plans reviewed, Kenya’s national nutrition plan stood out as an exemplar for its detailed budget lines for its monitoring and evaluation framework. ^i^ Nearly all components of the framework (except for equipment and supplies and ad hoc category) was specifically captured in the costed plan (Please see Table 1). The plan covers the full nutrition data value chain, including data use activities and also includes cost estimates for M&E related activities outside of the health sector, such as education and social protection. In sum, the plan allocates approximately 6% of the total estimated resource need for nutrition towards data and information systems.

**Table 1.** Kenya national nutrition action plan.

Nutrition data component	Total cost of M&E section of plan	Examples of line items included
1. Periodic data collection	27%	Major nutrition-focused surveys including SMART, MIYCN KAP, and other coverage surveys
2. Administrative/routine systems	23%	Activities focused on strengthening the routine HMIS systems, and integration of data systems for nutrition services delivered through HIV and TB programs
3. Information synthesis & decision making	28%	Development of nutrition dashboards, scorecards, or other electronic data visualization tools, as well as utilization of nutrition information to inform program quality improvement
4. Ad hoc data collection	0%	
5. Planning	9%	Reviewing and updating the Kenya M&E framework and to support the development and progress of other multi-year plans
6. Measurement innovations	2%	Investments in emerging technologies for nutrition assessment and diagnostics for HIV/TB patients
7. Human resources/capacity building	11%	Develop capacity for use of mHealth systems at the community level
8. Equipment/infrastructure	0%	

## Discussion

Nutrition data and information systems play a critical role in ensuring valid, reliable, and timely nutrition data are available, accessible, and used by key nutrition stakeholders to inform decision-making. However, our review revealed that there is a strong need for tools to support countries efforts in planning and costing NDIS. Countries need to develop comprehensive nutrition data plans to ensure prioritization, coherence and coordination of NDIS investments with nutrition relevant sectors, taking a long-term perspective. These data plans should be ideally developed with investment cases so that the latter can function as a guide to secure needed funding.

For the development of NDIS plans, countries may benefit from technical and financial support. For instance, countries may need support for developing the plan, costing it, ensuring relevant activities throughout the data value chain are considered. Financial support could be catalytic for fiscally constrained governments because strengthening NDIS could cost a substantial share of the total cost of the national nutrition plan (e.g. 6% in the case of Kenya) and generally tends to be underprioritized. For these reasons, key events like the N4G Summit provide an opportunity for the global community to make commitments to nutrition data systems and thereby support countries in their respective journeys.

Given that this is an area of limited research, we also wanted to share some reflections and questions that came up during our review, which may warrant further research and discussions-

- What would be an appropriate cost benchmark for national nutrition plans data related activities?
- How to ensure that NDIS is being systematically incorporated in relevant sectoral plans and budgets? What guidance could be provided to countries on how best to integrate NDIS within existing systems set up by relevant Ministries and departments such as the Statistical office, Health Ministry, Agriculture Ministry among others?
- As service delivery activities are increasingly being managed at the local level, information needs are getting localized as well as financial flows. When developing and costing NDIS plans, what should be the cost-sharing between the national and sub-national levels?
- How to integrate the costing and budgeting of NDIS within existing annual planning and budgeting processes?
- How can donors better align with country governments when thinking about financing sources for nutrition plans especially for NDIS costs?
- How should countries cost for key surveys such as the DHS and Multiple Indicator Cluster Surveys (MICS) that provide critical nutrition data but are costed and financed through other sectors or sources? More broadly, given that nutrition relies on data from broader systems, how should the NDIS approach the costing of these components?
- It is often argued that when it comes to allocation of scarce resources, it is hard to prioritize data systems over programmatic implementation. What advocacy efforts can help make the case that data funding can support countries to better plan, target and deliver their nutrition programs, and thus make their resources go further?

## Data availability

### Underlying data

DANS: How are countries planning for costs of nutrition data and information systems?

https://doi.org/10.17026/dans-xap-jq2v ( Manorat *et al*., 2020).

This project contains the file ‘N4G_Plans_Analysis_to share’. (This file includes the full list of national nutrition plans that were included in the analysis as well as individual country level data for the 19 countries that had costed nutrition plans in addition to costed NDIS related activities. For each country, the document includes a summary of the total plan costs, breakdown costs of NDIS activities, and percent of budg *et al*located towards nutrition data.)

### Extended data

DANS: How are countries planning for costs of nutrition data and information systems?

https://doi.org/10.17026/dans-xap-jq2v ( Manorat *et al*., 2020).

- Appendix 1. List of Stakeholders consulted (PDF).
- Appendix 2. Brief description of framework components with select examples from national nutrition plans (PDF).
- Appendix 3. List of national nutrition plans reviewed (PDF).

Data are available under the terms of the Creative Commons Zero “No rights reserved” data waiver (CC0 1.0 Public domain dedication).

## Notes

^i^Please note that we reviewed the draft version of the 2018–2022 Kenya Nutrition Action Plan (KNAP).
