# Supplementary material for: How are countries planning for costs of nutrition data and information systems?
Source: Gates Open Res. 2020 Jun 16;4:60. [Version 1] doi: 10.12688/gatesopenres.13145.1 (PMC7607158; doi:10.12688/gatesopenres.13145.1)
Supplement: Peer review history

# Peer review: Reviewer response for version 1

**DOI:** 10.21956/gatesopenres.14336.r29875

**Published:** 2020-11-02

**Authors:** Theresa Diaz

**Affiliations:** 1 Department of Maternal, Newborn, Child and Adolescent Health and Ageing, World Health Organization, Geneva, Switzerland

This is a well written comprehensive review of investments in data systems and capacity to use these data based on national nutrition plans. The authors clearly explain the nutrition data value chain and follow a framework that outlines the major components needed for establishing and maintaining nutrition data information systems. They also clearly explain the search criteria and number of documents that contain costed plans.

 They also highlight the one costed plan, Kenya, that contained a budget for the different components a full nutrition nutritional data information system. But it should be noted that Kenya is one of the premier countries in the health data collaborative that worked hard to have a one monitoring and evaluation plan for the country aligning donors, partners and health programs.   Details can be found here https://www.healthdatacollaborative.org/where-we-work/kenya/. This may be in part why the nutrition data information plan is so well documented.

 The major limitation of this study is that resource allocation and budgeting for national health information systems are normally part of national monitoring and evaluation plans. Although specific health programs should allocate portions of their budget toward data collection, analysis interpretation, it is difficult to determine if the funding in the nutrition plan aligns or adds to the national monitoring and evaluation plans and budget.

 The questions at the end of the article address some of these issues, specifically how NDIS is being systematically incorporated into in relevant sectorial plans and existing data systems and how countries cost for key surveys and how financing of these may be from donors and other sectors.  

 The question on “What would be an appropriate cost benchmark for nutrition plans?” really needs to address what the gaps are in the overall monitoring and evaluation plans and budget for the country. Would it not be better for national population based surveys, health information management systems basic infrastructure, capacity building etc. to be funded by the monitoring and evaluation budget and that specific health areas focus more on information synthesis and decision making?

 If there is any way for the authors can also conduct a search of the monitoring and evaluation plans and review their costing it would greatly contribute to this analysis and help better assess if the nutrition data information costs in the nutrition plans are well aligned.

Is the work clearly and accurately presented and does it cite the current literature?

Yes

If applicable, is the statistical analysis and its interpretation appropriate?

Not applicable

Are all the source data underlying the results available to ensure full reproducibility?

Yes

Is the study design appropriate and is the work technically sound?

Partly

Are the conclusions drawn adequately supported by the results?

Yes

Are sufficient details of methods and analysis provided to allow replication by others?

Yes

Reviewer Expertise:

I am the Unit Chief for Epidemiology and Monitoring and Evaluation for the Maternal Newborn Child and Adolescent Health and Ageing Department at WHO. I have over 30 years experience in surveillance and monitoring and evaluation including use of routine health information systems. I am not an expert in nutrition.

I confirm that I have read this submission and believe that I have an appropriate level of expertise to confirm that it is of an acceptable scientific standard.

# Peer review: Reviewer response for version 1

**DOI:** 10.21956/gatesopenres.14336.r28925

**Published:** 2020-07-28

**Authors:** Kendra Siekmans

**Affiliations:** 1 Nutrition International, Guelph, Canada

Consistent with the high quality and clarity of their previous work, the Results for Development team have conducted a useful and methodical analysis of the nutrition data and information system components included in SUN Movement member country national nutrition plans. SUN countries are committed to creating enabling environments for ending malnutrition in all its forms, including efforts to bring together stakeholders from multiple sectors and help each of those sectors recognize their role to play. National nutrition plans are an important tool to identify priority actions and who is responsible for delivering them, as well as how progress will be measured. As described by Renee Manorat and colleagues, at least 58 of the current 61 SUN countries have developed national nutrition plans and many of these are considered multi-sectoral, i.e. they represent the commitments to action by multiple government sectors toward the common goal of improving nutrition. 

 To better understand what nutrition data and information system components were included in these plans and how much these actions were expected to cost, Renee Manorat and colleagues conducted an in-depth review of 19 national nutrition plans which were publicly available and fully costed. Using a conceptual framework that describes the main components of nutrition data and information systems, the authors categorized the monitoring, evaluation and data activities in each country plan by these components, along with the cost associated with each activity. This allowed them to observe the range in types of nutrition data and information system activities identified in the plans as well as calculate the sum of their expected cost by each core and cross-cutting component.

 The results reveal that these 19 SUN countries vary widely in terms of the number of nutrition data and information system-related activities included in their national nutrition plan, the level of detail in which they are described and the costs associated with them. In fact, while the authors provide very nice data visualizations comparing the cost estimates, it is difficult to do much with these estimates, given the uncertainty underlying them. Put another way, the results show that few countries have identified (or included in their national nutrition plan) the specific activities needed to monitor and evaluate the results of their efforts to improve nutrition. This is, in itself, a very important and sobering finding.

 The authors of this study acknowledge this limitation in their approach - the summary of activities listed in the national nutrition plan does not fully represent the country's investments in nutrition data and information systems. However, a more extensive discussion on the possible reasons for this under-representation of nutrition data-related activities in these plans would be helpful to inform future technical and financial support. 

 One important factor that I recommend the authors consider further in this work is the political economy of nutrition, as described by Yarlini Balarajan and Michael Reich (2016) ^1^. For example, their description of the "institutional homelessness" of nutrition within government structures is an important factor influencing how effectively nutrition actors engage with other sectors in the planning and budgeting process. It would be interesting to explore this further with the data from this review, examining whether the position of nutrition within the government structure (e.g. supra-sectoral level such as the Prime Minister's Office vs. line ministry level such as within the Ministry of Health) influences the scale and types of nutrition data and information system actions outlined in the national nutrition plan.

 Another important factor to explore further is the tension between working with existing data and information systems vs. aspirational goals of identifying the data needed and seeking to find ways to collect them. The finding that only one country plan included measurement innovations suggests there is little vision for how data and information systems can be strengthened and expanded to better support nutrition policy and programme decision-making. DataDENT and National Information Platforms for Nutrition (NIPN) have provided leadership in this regard by working with countries to first identify the policy questions they have and then looking at what data are needed to answer them. What learning has come from those initiatives that could help put the findings of this review in context and further inform the recommendations made to strengthen these plans?

 The results also highlight the complex journey that SUN member countries find themselves on, with few exemplars to follow in terms of successful national multisectoral nutrition approaches. While the development and ratification of a national multi-sectoral nutrition plan is an achievement in itself, there is a need to continue learning and strengthening the content of these plans with each revision. Six of the plans reviewed in this study ended in the year 2018 or earlier and may have been the very first national nutrition plan developed in that country. It would be interesting to see whether countries with newer or updated plans were more likely to include costed nutrition data and information system activities.

 I recommend that in the Discussion section, the authors consider placing their results in the context of other research that has been done recently. For example, MQSUN+’s review of the quality of SUN national nutrition plans ^2^ and SPRING’s Pathways to Better Nutrition case studies from Nepal and Uganda ^3^ are two relevant reviews. I also wonder whether the discussion could include some additional reflection on the challenges experienced to date by SUN countries in the process of costing their nutrition plans, given that so many country plans were not fully costed and that even those with costed plans often did not have their nutrition data-related activities disaggregated to a level that made it possible to categorize them.

 Balarajan and Reich (2016) argue that the nutrition community's limited ability to clearly demonstrate results continues to hamper its efforts to rise on the political agenda. They suggest that greater attention to and investment in rigorous monitoring and evaluation as well as routine information systems is required to demonstrate clear progress and develop a compelling narrative that makes championing nutrition a more attractive option for politicians. The results of this study provide a clear wake-up call to global and country-level nutrition actors alike - increased technical and financial investment is needed to support countries in identifying and budgeting for the nutrition data and information systems actions that will enable them to track progress, adjust their programmes where needed, and clearly demonstrate progress towards their goals.

Is the work clearly and accurately presented and does it cite the current literature?

Partly

If applicable, is the statistical analysis and its interpretation appropriate?

Yes

Are all the source data underlying the results available to ensure full reproducibility?

Yes

Is the study design appropriate and is the work technically sound?

Yes

Are the conclusions drawn adequately supported by the results?

Yes

Are sufficient details of methods and analysis provided to allow replication by others?

Yes

Reviewer Expertise:

Multi-sectoral nutrition programme monitoring & evaluation in low- and middle-income countries; SUN Movement; infant and young child feeding; micronutrient deficiencies

I confirm that I have read this submission and believe that I have an appropriate level of expertise to confirm that it is of an acceptable scientific standard.

## References

[1] : Political economy challenges in nutrition. *Globalization and Health*.2016;12(1) : 10.1186/s12992-016-0204-6 10.1186/s12992-016-0204-6 27816061PMC5097844

[2] Review of SUN Country National Nutrition Plans. *MQSUN*.2020; Reference source

[3] : Recommendations for multi-sector nutrition planning: Cross-context lessons from Nepal and Uganda. Reference source
